# Profile of cardiac lipid metabolism in STZ-induced diabetic mice

**DOI:** 10.1186/s12944-018-0872-8

**Published:** 2018-10-09

**Authors:** Wenjie Li, Min Yao, Ruonan Wang, Yun Shi, Lianguo Hou, Ziyuan Hou, Kaoqi Lian, Nan Zhang, Yaqi Wang, Weiwei Li, Wei Wang, Lingling Jiang

**Affiliations:** 1 Department of Biochemistry and Molecular Biology, The Key Laboratory of Neurobiology and Vascular Biology, China Administration of Education, Hebei Medical University, No. 361 Zhongshan East Road, Shijiazhuang, 050017 China; 2 Anyang Center for Disease Control and Prevention, No. 01 Ziyou Road, Anyang, 455000 Henan Province China

**Keywords:** Diabetic myocardial disease, Lipotoxicity, Non-targeted metabolomics, Lipid metabolomics

## Abstract

**Background:**

Lipotoxicity contributes to diabetic myocardial disease. In this study, we investigated the lipid species contributing to lipotoxicity and the relationship with peroxisomal β-oxidation in the heart of diabetic mice.

**Methods:**

Male C57BL/6 mice were randomly divided into a Diabetic group (intraperitoneal injection of STZ) and a Control group (saline). Cardiac function indexes [ejection fraction (EF%) and fractional shortening (FS%)] were evaluated by echocardiography. Morphological changes in the myocardial tissues and mitochondria were assessed by electron microscopy following hematoxylin and eosin staining. Blood myocardial injury indexes and lipids were measured using an automatic biochemical analyzer. Cardiac ATP levels were analyzed using a commercially available kit. mRNA levels of glucose transporter 4 (GLUT4), fatty acid binding protein 3 (FABP3), palmitoyl transferase 1α (CPT-1α), acyl-CoA oxidase 1 (AOX1), D-bifunctional protein (DBP), 3-ketoacyl-CoA thiolase A (THLA), uncoupling protein (UCP) 2 and UCP3 were investigated by quantitative reverse-transcription polymerase chain reaction. FABP3 protein expression was analyzed by Western blotting. Non-targeted metabolomics by LC-MS/MS was applied to evaluate profile of lipid metabolism in heart.

**Results:**

Compared with controls, EF% and FS% were significantly reduced in diabetic mice. Furthermore, blood myocardial injury indexes and lipids, as well as myocardial mitochondrial cristae fusion were significantly increased. In the diabetic heart, GLUT4 expression was decreased, while expression of FABP3, CPT-1α, AOX1, DBP, THLA, UCP2 and UCP3 was increased, and ATP levels were reduced. In total, 113 lipids exhibited significant differential expression (FC > 2, *P* < 0.05) between the two groups, with sphingolipid metabolism identified as the top-ranking affected canonical pathway. In the diabetic heart, long-chain hydroxyl-acylcarnitines (8/8) and acylcarnitines (6/11), triglycerides (2/5), and diacyglycerol (3/7) were upregulated, while very long-chain polyunsaturated fatty acids (PUFAs) (5/6) including eicosapentaenoate, docosahexaenoate, phosphocholine (11/19), lysophosphocholine (5/9), phosphoethanolamine (7/11), lysophosphoethanolamine (7/10), phosphatidylglycerol (6/8), phosphoserine (6/8), phosphatidylinositol (2/2), phosphatidic acid (1/1), lysophosphatidic acid (1/1) and sphingomyelin (6/6) were downregulated.

**Conclusions:**

Our data suggest that the increase in toxic lipid species and decreased in PUFAs undergoing peroxisomal β-oxidation, combined with the reduction in phospholipids cause mitochondrial injury and subsequent uncoupling of phosphorylation and ATP deficiency; thereby leading to diabetic heart dysfunction.

## Background

Diabetic cardiomyopathy is the primary cause of morbidity and mortality in patients with diabetes [1–4], and is being increasingly recognized as a metabolic disease caused predominantly by lipotoxicity [5]. Under normal physiological conditions, approximately 70% of the ATP generated in the heart occurs through oxidation of fatty acids (FAs). Following entry of FAs into the cell via FA transporters located on the cell membrane, a CoA group is added, and the long-chain fatty acyl-CoA is converted to an acyl-carnitine. The resulting long-chain fatty acyl-carnitine then crosses the inner mitochondrial membrane, where FA oxidation occurs in the mitochondrial matrix [6].

In the heart from animals or humans with diabetes mellitus, there is a shift from glucose utilization to almost complete reliance on FAs as the energy source [6]. Therefore, the myocardium absorbs large amounts of FAs via CD36 molecules, which are integral membrane proteins that function as FA translocases. These FAs are then transferred into the mitochondria by cardiac fatty acid binding protein (FABP)3, which is upregulated in the diabetic heart of rat [7]. The expression of mitochondrial FA oxidation enzymes, such as carnitine palmitoyl transferase 1α (CPT-1α), is increased [8, 9]. However, some FAs [10], fatty acid acylcarnitine [11], triglycerides (TG) [12], and diacylglycerol (DG) [13] are increased, leading to impairment of mitochondrial structure and function, as well as cardiac function. In addition to the mitochondria, peroxisomes are also subcellular organelles involved in FA degradation. Long and medium chain-length FAs are well known as substrates for both mitochondrial and peroxisomal β-oxidation, although very long-chain FAs and mono- or polyunsaturated FAs (PUFAs) undergo β-oxidation only via the peroxisomal pathway to generate shorter chain acyl-CoA molecules for further β-oxidization to produce ATP via the mitochondrial pathway. However, identity of species related to the FAs undergoing peroxisomal β-oxidation among these accumulated lipid metabolites remains to be fully clarified.

Due to methodological issues, previous studies have not revealed the identity of lipids that play a role in lipotoxicity, although Han et al. [14] observed alterations of myocardial cardiolipin in diabetic heart of mice with a shotgun lipidomics study. This method involves ionization of lipid components by the ion source without chromatographic separation [15–17]; however, the ionization of low abundance and hard ionized lipid components was obviously inhibited, resulting in the inability of the related lipids to be detected [18]. Therefore, in the present study, a non-targeted metabolomics approach was used to investigate the potential of lipid metabolites to contribute to lipotoxicity and their relationship with peroxisomal FA oxidation in the diabetic heart.

## Methods

### Diabetes induction

Male C57BL/6 mice (aged 6–8 weeks, body weight approximately 20 g) were obtained from Beijing Vital River Laboratory Animal Technology Co. Ltd. (Certificate number 11400700128532). Mice were randomly divided into a Control group and Diabetic group (*n* = 6–8/ group).

For the diabetic model, the mice were intraperitoneally injected with a single dose (150 mg/kg body weight) of streptozotocin (STZ) (Sigma s0130, USA). Mice in the Control group received an equivalent volume of saline via the same route. Mice were considered diabetic with fasting blood glucose ≥16.7 mmol/L in two consecutive analyses conducted one week after STZ administration. After a further 6 weeks, fasting blood glucose measurements were repeated and body weight and cardiac function were evaluated. The mice were anesthetized by intraperitoneal injection of 20% Urethane (0.12 g/100 g, Sigma u2500, USA). Blood was collected immediately and stored at 37 °C for 30 min. Serum was then isolated by centrifugation at 1500 rpm for 15 min, at 4 °C fooled by collection of the supernatant. The serum was used for measurement of the myocardial injury index, total free fatty acid (T-FFA) content and other physiological indicators. The heart was removed immediately for ATP detection. Samples of cardiac tissue were stored at − 80 °C for quantitative real-time PCR and Western blot analyses, fixed in formaldehyde for hematoxylin-eosin (HE) staining, or fixed in glutaraldehyde for electron microscopy studies.

All animal experiment protocols were performed in accordance with the guidelines established by the Ethics Review Committee for Animal Experimentation (Hebei Medical University, Shijiazhuang, China).

### Echocardiographic assessment

Cardiac function was evaluated in the anesthetized mice (oxygen flow meters 0.5–1 L/min, Isoflurane flow meters 2–3%). Ultrasound examinations were carried out using a high resolution imaging system (Vevo 770, VisualSonics, Canada), equipped with a high-frequency ultrasound probe (RMV-707B, VisualSonics).

The settings for temporal resolution in M-mode imaging in this system were as follows: pulse repetition frequency, 8 kHz; axial resolution, 55 μm; lateral resolution, 115 μm; focal length, 12.7 mm; depth of field, 2.2 mm. M-mode images were obtained for measurements of left ventricular (LV) wall thickness, LV end-diastolic diameter (LVEDD), and LV end-systolic diameter (LVESD) (measures of LV dilation). Ejection fraction (EF) and fractional shortening (FS) were calculated as follows: EF% = LVEDV - LDESV/LVEDV × 100 and FS% = (LVEDD - LVESD/LVEDD) × 100 (measures of systolic function). To minimize variability of the data, cardiac function was assessed at a heart rate of 500–600 bpm.

### Determination of myocardial injury indexes in blood

Total creatine kinase (T-CK, KU/L), creatine kinase isoenzyme-MB (CKMB, KU/L), lactate dehydrogenase (LDH, KU/L), α-hydroxybutyrate dehydrogenase (α-HBDH, KU/L), myoglobin (Myo, ng/L), high sensitivity C reactive protein (Hs-CRP, mg/L) and homocysteine (HCY, mmol/L) were quantified using an automatic biochemical analyzer (AU 640 Medical System, Olympus, Japan) according to the manufacturer’s protocols.

Total free fatty acid (T-FFA, mmol/L) was assayed by colorimetry assay kits (NEFA, Nanjing Jiancheng Bioengineering Institute, Nanjing, China) following the manufacturer’s instructions. Total cholesterol (T-chol, mmol/L) and total triglycerides (T-TG, mmol/L) were quantified using an automatic biochemical analyzer (AU 640 Medical System, Olympus) according to the manufacturer’s protocols.

### HE staining

To investigate pathological changes in tissue structure, formalin-fixed heart samples were imbedded in paraffin and sections (thickness 4 μm) were prepared. The myocardial morphology was evaluated following HE staining according to standard procedures. Images were captured with a PictureFrame computer software (LAS V4.3) under a microscope (DM6000; Leica, Germany).

### Transmission electron microscopy

Glutaraldehyde-fixed cardiac tissue samples were washed in 0.1 M phosphate-buffered saline (PBS), post-fixed with 1% osmium tetroxide for 1 h, dehydrated in acetone, and embedded in resin. Sections (thickness 60 nm) were prepared and stained with 1% uranyl acetate and 0.4% lead citrate. Mitochondrial structure was assessed by transmission electron microscopy (TEM) using a JEOL JEM-1230 (JEOL, Japan).

### ATP determination

ATP (mol/g protein) was measured in the cardiac tissue samples using ATP assay kits (Nanjing Jiancheng Bioengineering Institute, Nanjing, China) according to the manufacturer’s instructions.

### Quantitative real-time PCR analysis

Total RNA was extracted from the cardiac tissue using the Total RNA Kit (OMEGA, UK). The cDNA was synthesized using the M-MLV First Strand Kit (Invitrogen, USA) and qRT-PCR analysis was carried out using the SYBR Green Master Mix (Thermo, USA) with the ABI 7500 Fast system (Life Technologies, USA). Expression of the following genes was analyzed: glucose transporter GLUT4, fatty acid transporter FABP3, carnitine palmitoyl transferase 1α (CPT-1α, a key enzyme for mitochondrial fatty acid β-oxidation), and the peroxisomal fatty acid β-oxidation enzymes acyl-CoA oxidase 1(AOX1), D-bifunctional protein (DBP), 3-Ketoacyl-CoA thiolaseA (THLA) and uncoupling protein (UCP)2, and UCP3; 18S rRNA served as a reference gene. The following gene-specific primers were used:

- 18S rRNA-F: 5^′^ -CGCCGCTA GAGGTGAAATTC-3^′^
- 18S rRNA-R: 5^′^-CCAGTCGGCATCGTTT ATGG-3^′^;
- CPT-1α-F: 5^′^-TACCTGCTTTGAAATGGGTGC-3^′^
- CPT-1α-R: 5^′^-AAGTGCACATGAGAGGTTGGA-3^′^;
- FABP3-F: 5^′^-AGTCACTGGTGACGCTGGACG-3^′^
- FABP3-R: 5^′^-AGGCAGCATGGTGCTGAGCTG-3^′^;
- GLUT4-F: 5^′^-GGGGTTATCAATGCCCCACA-3^′^
- GLUT4-R: 5^′^-GAAGATGGCCACGGAGAGAG-3^′^;
- THLA-F: 5^′^-TTCTCCAGGACGTGAGGCTA-3^′^
- THLA-R: 5^′^-GGCTCCTGGCTCAAGAACAT-3^′^;
- DBP-F: 5^′^-GAAAGGCGGAAAAGCAGTGG- 3^′^
- DBP-R:5^′^-AATGTGTCCAGTGCCGTCTT-3^′^;
- AOX1-F: 5^′^-CAGAACGGAAGCTGGAGTGT-3^′^
- AOX1-R: 5^′^-CCTGGCCGCCTATGTGTATT-3^′^;
- UCP2-F: 5^′^- CTCAGAAAGGTGCCTCCCGA-3^′^
- UCP2-R:5^′^- ATCGCCTCCCCTGTTGATGTGGTCA-3^′^;
- UCP3-F: 5^′^- GCACTGCGGCCTGTTTTG -3^′^
- UCP3-R: 5^′^- ACCCTCTGTGCGCACCATAGTCA-3^′^

The thermal cycling program was as follows: 10 s denaturation at 95 °C followed by 40 cycles of 5 s denaturation at 95 °C, 30 s annealing at 65 °C, and 20 s extension at 72 °C. The relative expression level of each gene was determined using the 2^−ΔΔCt^ method.

### Western blot analysis

The cardiac tissue was added to protein lysis buffer (50 mmol/L Tris-HCL pH 7.5, 150 mmol/L NaCL, 2 mmol/L EDTA, 1% TritonX-100, protease inhibitor) and homogenised on ice for 3–5 min. Total proteins were obtained by centrigugation of the homogenate at 12,000 rpm for 30 min at 4 °C. The proteins were resolved by SDS-PAGE (15% gel, 200 V for approximately 40 min) and then transferred to nitrocellulose membranes (semi-dry transfer at 20 V for 30 min). FABP3 and β-actin proteins were probed overnight at 4 °C with a polyclonal antibody (FABP3 15KDa anti-rabbit,β-actin 43KDa anti-rabbit, Santa Cruz, 1:1000). After removal of the primary antibody, the blots were washed with PBS/Tween-20, followed by incubation with a peroxidase-conjugated goat anti-rabbit IgG secondary detection antibody (Santa Cruz, 1:5000) at room temperature for 1 h. After washing, the immunoreactive bands were Visualizer with an ECL detection system and quantified by scanning with a Fusion Image Dock Station (Fusion FX5, Vilber Lourmat, France).

### LC-MS/MS analysis

#### Sample preparation

Cardiac tissue (100 mg) was placed in 1 mL pre-cooled (− 20 °C) 80% methanol, subjected to ultrasonic disruption and placed on ice for 20 min. The tissue was then centrifuged at 10,000×*g* for 10 min at 4 °C and 600 μL supernatant was freeze-dried and stored at − 80 °C. Before the experiment, the extracts were dissolved in 100 μL 80% methanol, and 5 μL was used for LC-MS/MS analysis using a LC system (Nexera X2 system, Shimadzu, JPN) consisting of a vacuum degasser, an autoinjector, and a triple quadruple mass spectrometer (MS/MS) (Triple TOF 5600+, AB Sciex, USA) equipped with analyst software MarkerView 1.2.1 for data acquisition and calibration.

Metabolites were separated on an Agilent ZORBAX Eclipse Plus C18 column (2.1 × 100 mm, 3.5 μm) at a flow rate of 0.5 mL/min. The mobile phase A comprised 0.1% formic acid/water and the mobile phase B comprised 0.1% formic acid/acetonitrile. The gradient of A was as follows: 98% (0–1 min), 98–10% (1–13 min), 10% (13–16 min), and 10–98% (16–16.1 min). The sample injection volume was 5 μL and the total run time was 16.1 min. The mass spectrometer equipped with an electrospray ionization source (ESI) at the resolution and in positive ion mode. The desolvation temperature was set to 500 °C and the source temperature was 120 °C. The desolvation and cone gas flow were set to 600 and 50 L/H, respectively. The positive ion mode was set to 3.0 kV. The sampling cone and extraction cone were set to 27 eV and 4 eV, respectively. The quadrupole analyzer ranged from 50 to 1500 m/z.

#### Statistical analysis

For the metabolomic analysis, LC-MS/MS data (metabolite peak intensity) were analyzed using MetaboAnalyst (v3.0) (http://www.metaboanalyst.ca/) feature of the statistical package R (v2.14.0). These data were scaled using the Pareto scaling feature and differences between the Control and Diabetic groups were analyzed using Student’s *t*-test. *P* < 0.05 was considered to indicate statistical significance. Multivariate analysis [principal component analysis (PCA) and partial least squares discriminant analysis (PLS-DA)] was performed using the MultiQuant software in MetaboAnalyst 3.0. PLS-DA was determined using the variable importance in projection (VIP) analysis. Compound identification was conducted using the METLIN and HMDB databases. Pathway analysis was performed by the Pathway Analysis features in MetaboAnalyst 3.0 (Impact > 0). All heat maps were generated using the heat map analysis feature in MetaboAnalyst v3.0.

All other data were expressed as mean ± standard deviation (SD). Differences between the Control and Diabetic groups were compared using Student’s *t*-test. *P* < 0.05 was considered to indicate statistical significance.

## Results

### Cardiac function and lipid metabolism abnormity in diabetic mice

As shown in Table 1, a significant increase in fasting glucose levels[fold change (FC) 4.9] and a significant decrease in body weight (FC 0.8) was observed in the Diabetic group compared to those in the Control group mice. In the Diabetic group, this was accompanied by significant increases in blood lipids including T-FFA (FC 2.6), T-chol (FC 1.3), T-TG (FC 2.2) and blood indicators of myocardial function such as T-CK (FC 9), CKMB (FC 2.5), LDH (FC 1.5), α-HBDH (FC 1.8), Myo (FC 1.4), Hs-CRP (FC 2) and HCY (FC 1.3). There were also significant decreases in the EF% (FC 0.7) and FS% (FC 0.7) in the Diabetic group compared with those in the Control group. These results indicated abnormalities in lipid metabolism and cardiac function in diabetic mice.

**Table 1:** Animal characteristics of mice

	Control (*n* = 8)	Diabetic (*n* = 8)	*P* value
Body weight, g	25.16 ± 0.88	16.30 ± 1.16	0.002
Ejection fraction,%	57.65 ± 11.52	42.00 ± 12.27	0.040
Fractional shortening,%	38.30 ± 9.00	27.95 ± 8.28	0.030
In blood			
Fasting glucose, mmol/L	5.49 ± 0.46	26.61 ± 1.14	0.002
Total free fatty acid, mmol/L	0.50 ± 0.19	1.32 ± 0.56	0.002
Total cholesterol, mmol/L	2.15 ± 0.40	2.81 ± 0.31	0.030
Total triglyceride, mmol/L	0.75 ± 0.21	1.64 ± 0.55	0.020
Total creatine kinase, KU/L	0.44 ± 0.15	0.83 ± 0.16	0.040
Creatine kinase isoenzyme-MB, KU/L	0.42 ± 0.11	1.04 ± 0.17	0.002
Lactate dehydrogenase, KU/L	0.65 ± 0.28	1.01 ± 0.25	0.040
α-hydroxybutyrate dehydrogenase, KU/L	0.31 ± 0.11	0.55 ± 0.13	0.002
Myoglobin, ng/L	6.86 ± 2.12	23.67 ± 3.44	0.001
High sensitivity C reactive protein, mg/L	0.20 ± 0.06	0.40 ± 0.140	0.010
Homocysteine, mmol/L	10.73 ± 1.77	13.67 ± 2.42	0.040

Data are means ± SD. *P* vs. Control

#### Mitochondrial structure abnormalities and decreased ATP levels in cardiac tissue in diabetic mice

**Fig. 1:**
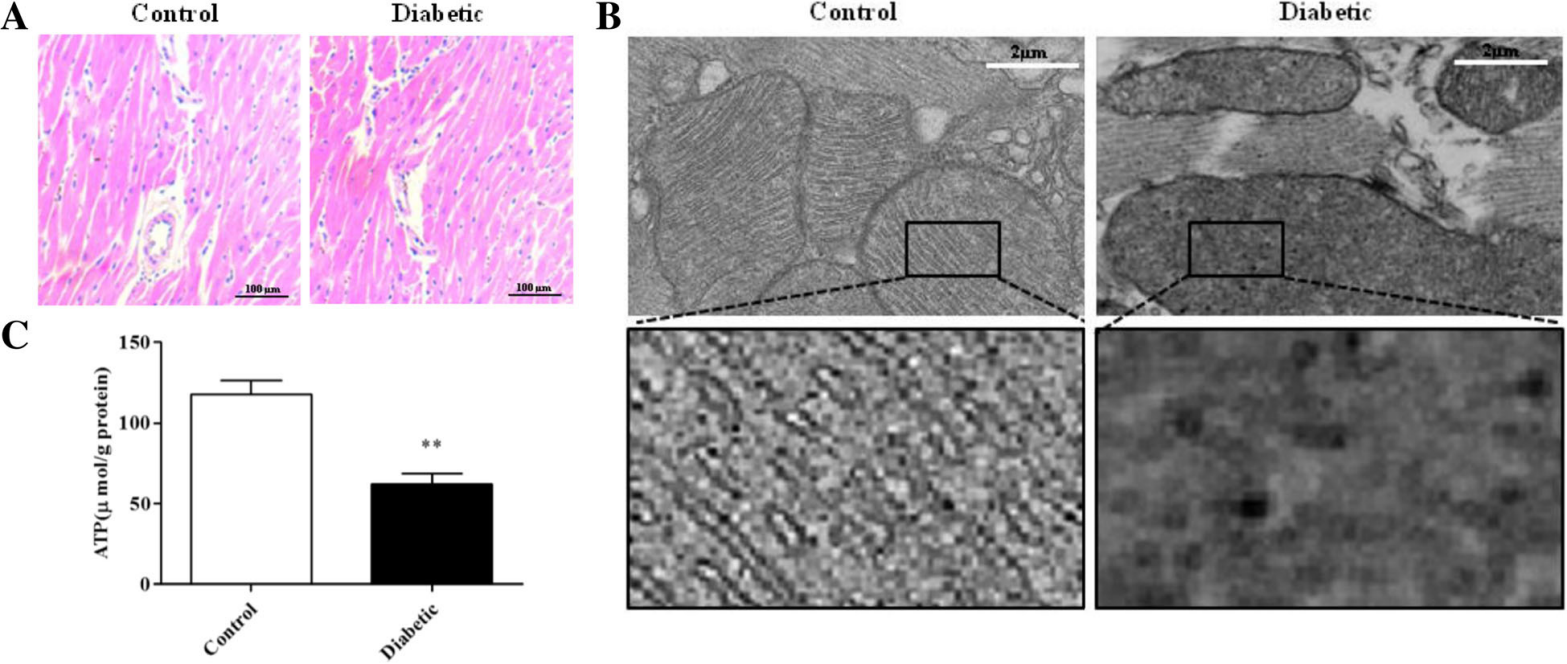
Mitochondrial structure and ATP content in mouse cardiac tissue. **a** HE staining images of cardiac tissue. **b** TEM images of mitochondrial structure (5000×), box: 7× magnification of the original image. **c** ATP content (*n* = 6). Data represent the means ± SD (*P < 0.05 vs. Control)

**Fig. 2:**
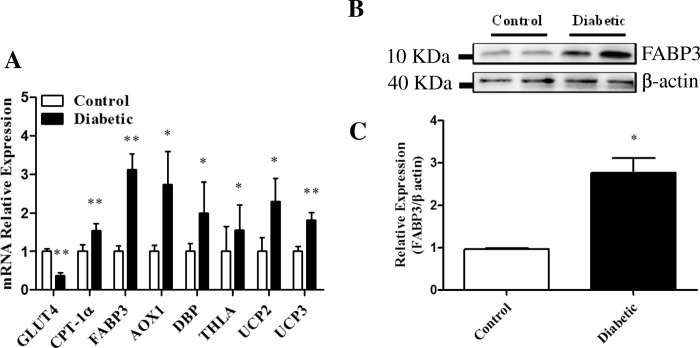
Expression of glucose utilization and fatty acid metabolism genes in cardiac tissue. **a** mRNA levels of GLUT4, CPT-1α, FABP3, AOX1, DBP, THLA, UCP2 and UCP3 (*n* = 6). **b** Western blot analysis of FABP3 and **c** its band intensity (*n* = 6).Data represent the means ± SD (*P < 0.05 or **P < 0.01 vs. Control)

Although HE staining showed no pathological changes in the cardiac tissue in the Diabetic group compared with that in the Control group (Fig. 1a), TEM images showed more mitochondrial cristae fusion (Fig. 1b). Furthermore, the mRNA levels of UCP2 and UCP3 were increased (Fig. 2a) in the Diabetic group. In accordance with these findings, reduced levels of ATP (Fig. 1c) were detected in the cardiac tissue in the Diabetic group.

#### Expression of genes involved in fatty acid-degradation were increased in the cardiac tissue of diabetic mice

As shown in Fig. 2, the mRNA expression of GLUT4 decreased, while expression of FABP3 increased at both the mRNA and protein levels increased in the cardiac tissue of diabetic mice. In addition, the mRNA expression of CPT-1α, AOX1, DBP and THLA also increased in the cardiac tissue of diabetic mice. These results indicated that glucose utilization was reduced and β-oxidation of FAs was increased both in the mitochondria and peroxisomes in the cardiac tissue of diabetic mice.

#### Changes in lipid metabolism in the cardiac tissue of diabetic mice

**Fig. 3:**
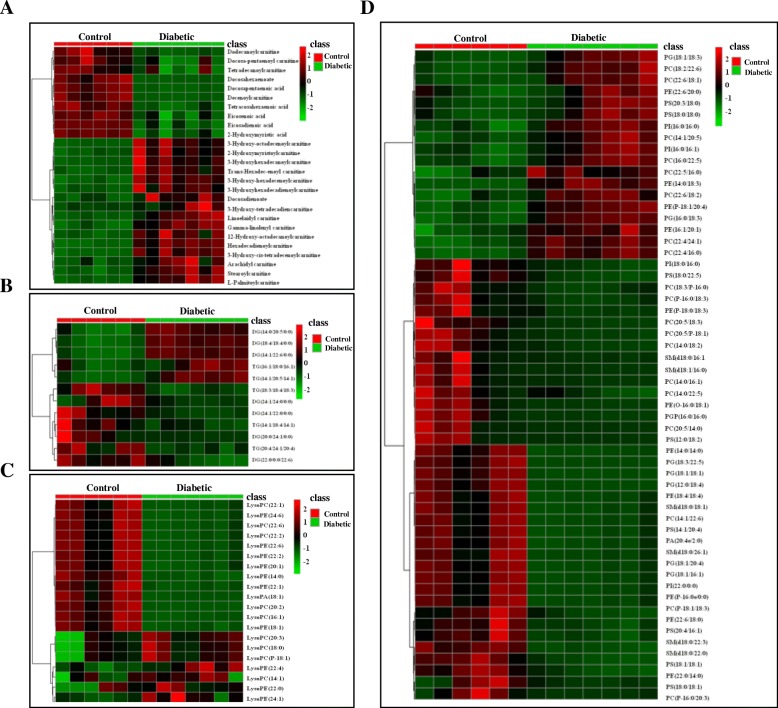
Heat map of the most abundant *t*-test significant lipid metabolites in mouse cardiac tissue. Each cardiac tissue sample (6 control samples shown in red, 7 diabetic samples are shown in green) is represented by a single column, and each column represents a different metabolite: **a** fatty acid, hydroxyacyl-carnitine and fatty-acyl-carnitine. **b** DG and TG. **c** Lysophospholipids and **d** PE, PC, PG, PS, SM in one stool sample. Red indicates higher abundance, while green indicates lower abundance

**Fig. 4:**
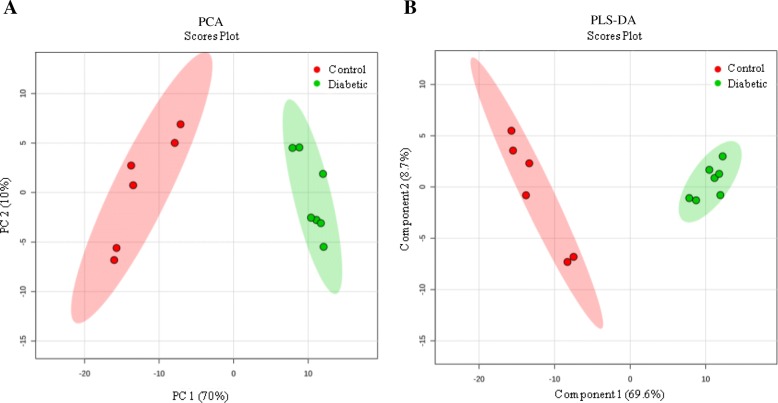
PCA (**a**) and PLS-DA (**b**) plots of the metabolome of mouse cardiac tissue. The control (red) and diabetic (green) cardiac tissue samples were used to determine discrimination between the Control and Diabetic heart samples

LC-MS/MS analysis revealed a total of 196 endogenous metabolites in the cardiac tissues of mice in the Control and Diabetic groups, of which 113 lipids exhibited significant differential expression (FC > 2, *P* < 0.05) (Fig. 3 and Table 2). PCA and PLS-DA analyses showed good separation between the data for the two groups (Fig. 4), indicating marked differences in the metabolic characteristics of the cardiac tissue in the two groups. Furthermore, these results confirmed that the data obtained in present study were representative and with good repeatability.

**Table 2:** The Significantly different metabolites in heart of mice

Class	Metabolites	FC(Diabetic/Control)	log2(FC)	Vip Value	*p* Value
Hydroxyl-fatty-acyl-carnitine	2-Hydroxymyristoylcarnitine(18:0)	3.182	1.66993	1.133	0.0472
	3-Hydroxy-cis-tetradecenoylcarnitine(14:1)	10.212	3.35219	1.174	0.0308
	3-Hydroxy-tetradecadiencarnitine(14:2)	2.552	1.35163	1.230	0.0163
	3-Hydroxyhexadecanoylcarnitine(16:0)	11.681	3.54609	1.230	0.0323
	3-Hydroxy-hexadecenoylcarnitine(16:1)	2.500	1.32193	1.188	0.0433
	3-Hydroxyhexadecadienoylcarnitine(16:2)	2.721	1.44414	1.146	0.0450
	3-Hydroxy-octadecenoylcarnitine(18:1)	3.077	1.62152	1.139	0.0465
	12-Hydroxy-octadecanoylcarnitine(18:0)	3.293	1.71940	1.061	0.0467
Fatty-acyl-carnitine	Decenoylcarnitine(10:1)	0.033	−4.93669	1.324	< 0.0001
	Dodecanoylcarnitine(12:0)	0.202	−2.30685	1.335	< 0.0001
	Tetradecanoylcarnitine(14:0)	0.481	−1.05589	1.076	< 0.0001
	Docosa-pentaenoyl carnitine(22:5)	0.455	− 1.13488	1.247	0.0079
	L-Palmitoylcarnitine(16:0)	2.421	1.27560	1.062	0.0107
	trans-Hexadec-enoyl carnitine(16:1)	2.132	1.09221	1.112	0.0224
	Hexadecadienoylcarnitine(16:2)	2.594	1.37518	1.349	0.0630
	Stearoylcarnitine(18:0)	2.404	1.26544	1.050	0.0130
	Linoelaidyl carnitine(18:2)	2.053	1.03773	1.016	0.0182
	Gamma-linolenyl carnitine(18:3)	2.202	1.13881	1.123	0.0189
	Arachidyl carnitine(20:0)	0.429	−1.22219	1.095	0.0200
Fatty acid	Eicosenoic acid(20:1)	0.459	−1.12499	1.347	< 0.0001
	Eicosapentaenoic acid(20:5)	0.479	−1.06212	1.143	< 0.0001
	Docosadienoate(22:2)	0.089	−3.48877	1.343	< 0.0001
	Docosahexaenoate(22:6)	0.067	−3.90785	1.322	< 0.0001
	Tetracosahexaenoic acid(24:6)	0.215	−2.21475	1.384	< 0.0001
	Docosapentaenoic acid(22:5)	2.084	1.05936	1.275	0.0079
Triglyceride (TG)	TG 14:1/18:4/14:1	0.365	−1.45418	1.152	0.0284
	TG 18:3/18:4/18:3	0.263	−1.92638	1.189	0.0024
	TG 20:4/24:1/20:4	0.350	−1.51450	1.046	0.0033
	TG 14:1/20:5/14:1	6.763	2.75766	1.295	< 0.0001
	TG 16:1/18:0/16:1	3.448	1.78576	1.065	0.0268
Diacylglycerol (DG)	DG 20:0/24:1/0:0	0.411	−1.28451	1.335	< 0.0001
	DG 22:0/0:0/22:6	0.109	−3.20398	1.089	0.0078
	DG 24:1/22:0/0:0	0.454	−1.13881	1.049	0.0006
	DG 24:1/24:0/0:0	0.354	−1.49621	1.206	0.0005
	DG 14:0/20:5/0:0	2.538	1.34369	1.372	< 0.0001
	DG 14:1/22:6/0:0	4.715	2.23726	1.393	< 0.0001
	DG 18:4/18:4/0:0	4.079	2.02822	1.395	< 0.0001
phospholipid					
Phosphatidylcholine (PC)	PC 14:0/16:1	0.135	−2.89414	1.026	0.0150
	PC 14:0/18:2	0.228	−2.13027	1.030	0.0094
	PC 14:0/22:5	0.409	−1.28924	1.091	0.0340
	PC 14:1/22:6	0.103	−3.27769	1.192	0.0014
	PC P-16:0/18:3	0.305	−1.71326	1.050	0.0121
	PC P-16:0/20:3	0.392	−1.35276	1.333	0.0003
	PC P-18:1/18:3	0.353	−1.50131	1.075	0.0236
	PC 18:3/P-16:0	0.411	−1.28155	1.043	0.0199
	PC 20:5/P-18:1	0.410	−1.28570	1.056	0.0258
	PC 20:5/18:3	0.110	−3.18412	1.712	0.0240
	PC 20:5/14:0	0.428	−1.22342	1.243	0.0147
	PC 14:1/20:5	2.075	1.05311	1.303	< 0.0001
	PC 16:0/22:5	2.316	1.21164	1.163	0.0023
	PC 18:2/22:6	6.391	2.67604	1.086	0.0056
	PC 22:4/16:0	2.178	1.12300	1.301	< 0.0001
	PC 22:4/24:1	2.018	1.01293	1.288	< 0.0001
	PC 22:5/16:0	2.358	1.23756	1.207	0.0011
	PC 22:6/18:1	2.065	1.04614	1.211	0.0012
	PC 22:6/18:2	5.212	2.38184	1.161	0.0019
Lysophosphatidylcholine (LysoPC)	LysoPC 16:1	0.203	−2.30217	1.254	< 0.0001
	LysoPC 20:2	0.072	−3.79286	1.282	< 0.0001
	LysoPC 22:1	0.130	−2.94823	1.201	0.0012
	LysoPC 22:2	0.122	−3.04002	1.213	0.0010
	LysoPC 22:6	0.128	−2.96532	1.208	0.0011
	LysoPC 14:1	2.016	1.01150	1.005	0.0243
	LysoPC 18:0	2.145	1.10098	1.023	0.0108
	LysoPC P-18:1	2.111	1.07793	1.030	0.0101
	LysoPC 20:3	2.254	1.17249	1.092	0.0055
Phosphoethanolamine (PE)	PE 14:0/14:0	0.028	−5.16792	1.276	< 0.0001
	PE P-16:0e/0:0	0.167	−2.58448	1.184	0.0016
	PE O-16:0/18:1	0.091	−3.46205	1.114	0.0091
	PE P-18:0/18:3	0.338	−1.56365	1.171	0.0232
	PE 18:4/18:4	0.122	−3.02998	1.213	0.0010
	PE 22:0/14:0	0.259	−1.94935	1.440	< 0.0001
	PE 22:6/18:0	0.303	−1.72072	1.312	< 0.0001
	PE 14:0/18:3	8.300	3.05311	1.423	< 0.0001
	PE 16:1/20:1	5.469	2.45128	1.352	< 0.0001
	PE P-18:1/20:4	3.854	1.94636	1.287	< 0.0001
	PE 22:6/20:0	2.035	1.02503	1.282	< 0.0001
Lysophosphatidylethanolamine (LysoPE)	LysoPE 14:0	0.465	−1.10434	1.158	0.0022
	LysoPE 18:1	0.315	−1.66585	1.268	< 0.0001
	LysoPE 20:1	0.150	−2.73314	1.232	0.0007
	LysoPE 22:1	0.209	−2.26093	1.258	< 0.0001
	LysoPE 22:2	0.190	−2.39890	1.237	0.0006
	LysoPE 22:6	0.122	−3.03386	1.213	0.0010
	LysoPE 24:6	0.116	−3.11103	1.248	0.0005
	LysoPE 22:0	2.026	1.01863	1.021	0.0326
	LysoPE 22:4	2.339	1.22589	1.214	0.0008
	LysoPE 24:1	3.518	1.81476	1.180	0.0048
phosphatidic acid (PA)	PA 20:4e/2:0	0.127	−2.97911	1.175	0.0019
Lysophosphatidic acid (LysoPA)	LysoPA 18:1	0.202	−2.30918	1.203	0.0011
Phosphatidylglycerol (PG)	PG 12:0/18:4	0.165	−2.59837	1.235	0.0006
	PGP 16:0/16:0	0.109	−3.19141	1.185	0.0210
	PG 18:1/16:1	0.168	−2.57628	1.297	< 0.0001
	PG 18:1/18:1	0.109	−3.20383	1.211	0.0010
	PG 18:1/20:4	0.110	−3.18713	1.207	0.0011
	PG 18:3/22:5	0.106	−3.24031	1.289	< 0.0001
	PG 16:0/18:3	6.018	2.58928	1.353	< 0.0001
	PG 18:1/18:3	2.525	1.33628	1.078	0.0058
Phosphatidylserine (PS)	PS 12:0/18:2	0.163	−2.61965	1.260	< 0.0001
	PS 14:1/20:4	0.117	−3.09238	1.189	0.0014
	PS 18:0/18:1	0.478	−1.06626	1.334	< 0.0001
	PS 18:0/22:5	0.394	−1.34426	1.137	0.0055
	PS 18:1/18:1	0.412	−1.27977	1.611	0.0279
	PS 20:4/16:1	0.274	−1.86632	1.293	< 0.0001
	PS 18:0/18:0	5.065	2.34056	1.257	< 0.0001
	PS 20:3/18:0	5.523	2.46545	1.230	0.0007
Phosphatidylinositol (PI)	PI 18:0/16:0	0.457	−1.12829	1.064	0.0211
	PI 22:0/0:0	0.130	−2.94505	1.203	0.0012
	PI 16:0/16:0	2.180	1.12433	1.211	0.0010
	PI 16:0/16:1	2.423	1.27679	1.176	0.0020
Sphingomyelin (SM)	SM d18:0/16:0	0.100	−3.32034	1.121	0.0127
	SM d18:0/16:1	0.303	−1.72072	1.058	0.0358
	SM d18:0/18:1	0.126	−2.98841	1.208	0.0010
	SM d18:0/22:0	0.329	−1.60549	1.055	0.0236
	SM d18:0/22:3	0.227	−2.14176	1.342	< 0.0001
	SM d18:0/26:1	0.099	−3.33914	1.202	0.0012

*FC* fold change, *VIP value* variable importance in the projection; *p* Value, from Student t test and vs. diabetic group; Metabolites, lipids displaying significant changes between control and diabetic heart (*P* < 0.05 and VIP > 1)

Among the 113 differentially expressed lipids detected in the cardiac tissue of diabetic mice, the lipids were FFAs, acyl-carnitines, hydroxyl-acyl-carnitines, TGs, DGs, phosphocholine (PC), phosphoethanolamine (PE), phosphatidylglycerol (PG), phosphoserine (PS), phosphatidylinositol (PI), phosphatidic acid (PA) lysoglycerophospholipids (LysoPC, LysoPE and LysoPA) and sphinomyelin (SM).

Compared with the Control group, all six differentially expressed FFAs were unsaturated very long-chain fatty acids (C20–C24) and all were significantly downregulated in the cardiac tissue of diabetic mice, with the exception of C22:2 FFA, which was upregulated. Eight differentially expressed hydroxyl-acyl-carnitines were identified, all of which were long-chain (C14–C18) and all were upregulated in the cardiac tissue of diabetic mice. Eleven differentially expressed acyl-carnitines were identified (4 downregulated and 6 upregulated), all of which were long-chain (C10–C20), with the exception of docosapentaenoyl carnitine (C22). Of the downregulated acyl-carnitines in the Diabetic group, the levels of two (C10:1 and C12:0) were a thirtieth and a fifth of the levels detected in the Control group, indicating increased mitochondrial β-oxidation of medium chain FAs in the diabetic heart.

Five differentially expressed TGs were identified in the cardiac tissue of diabetic mice, of which two TGs (with more long chain FA) were upregulated (FC 6.7 and 3.6) and three (more polyunsaturated FA) were downregulated. Seven differentially expressed DGs were identified (3 upregulated and 4 downregulated) in the cardiac tissue of diabetic mice. Among the four downregulated DGs, levels of the DG (22:0/0:0/22.6) were one tenth of the levels detected in the Control group, indicating increased peroxisomal β-oxidation in the diabetic heart.

Compared with the Control group, nineteen differentially expressed PCs (11 downregulated and 8 upregulated) and nine differentially expressed lysoPCs (5 downregulated and 4 upregulated) were identified in the cardiac tissue of diabetic mice. In addition, 11 differentially expressed PEs (7 downregulated and 4 upregulated) and 10 differentially expressed lysoPEs (7 downregulated and 3 upregulated) were identified in the cardiac tissue of diabetic mice. Eight differentially expressed PGs (6 downregulated and 2 upregulated) were identified in the cardiac tissue of diabetic mice. Eight differentially expressed PSs (6 downregulated and 2 upregulated) were identified in the cardiac tissue of diabetic mice. Four differentially expressed PIs (2 downregulated and 2 upregulated) were identified in the cardiac tissue of diabetic mice. One PA and one lysoPA as well as six SMs were downregulated in the cardiac tissue of diabetic mice compared with the levels detected in the Control group.

#### Pathway analysis in heart of diabetic mice

With the impact value threshold set at > 0, the result of the pathway analysis suggested that the key metabolites were involved in seven metabolic pathways (Table 3). Sphingolipid metabolism was the top-ranking impacted canonical pathway.

**Table 3:** Pathway analysis of key metabolites

Pathway	Total	Hits	Raw *p*	Impact
Sphingolipid metabolism	21	4	0.09957	0.27820
Glyoxylate and dicarboxylate metabolism	18	1	0.80423	0.09677
Pantothenate and CoA biosynthesis	15	1	0.74270	0.06122
Glycosylphosphatidylinositol(GPI)-anchor biosynthesis	14	2	0.34274	0.04390
Glycerolipid metabolism	18	1	0.80423	0.01920
Fatty acid elongation in mitochondria	27	1	0.91409	0.00370
Fatty acid metabolism	39	2	0.86411	0.00034

Total, total number of compounds in the pathway. Hits, the number of compounds that match with our experimental data. Raw *p*, original *p* value calculated from the enrichment analysis. Impact, pathway impact value calculated from pathway topology analysis

## Discussion

Hyperglycemia, hypertriglyceridemia, abnormalities of lipid metabolism and cardiac function injury are the main features of diabetic cardiomyopathy [19–21]. In STZ-induced diabetic animal models, elevated blood glucose and lipid levels are associated with cardiac contractile function deterioration and increased myocardial injury indexes in blood [19, 22]. In accordance with these reports, in the present study, cardiac EF% and FS% were reduced along with increased blood glucose and lipid levels in detected in STZ-induced diabetic mice.

Insulin deficiency causes decreased GLUT4 expression and subsequent reduction in glucose delivery and increased FA intake by cardiomyocytes as an energy source [23, 24]. These FAs in myocardial cells are then transported by the upregulated cardiac FABP3 [25] to the mitochondria for β-oxidation to generate ATP [26]. The enzymes involved in β-oxidation are highly regulated at the transcriptional level, and their expression is often associated with upregulation of fatty acid β-oxidation [27]. Furthermore, CPT-1α, which is the rate limiting enzyme in the process of mitochondrial β-oxidation, is transcriptionally upregulated in the diabetic heart to elevate the rate of mitochondrial β-oxidation for degradation of these FAs [28]. In accordance with these reports, in the present study, we also observed downregulation of GLUT4 expression in the cardiac tissue of diabetic mice, while FABP3 and CPT-1α expression was upregulated, indicating reduced glucose utilization and increased mitochondrial β-oxidation for degradation of these FAs, the majority of which were long-chain FA. However, although FA is dramatically increased [29], the substantial FFA uptake often exceeds the oxidative capacity of the mitochondria [27]. This leads to FA elevation and subsequent TG synthesis in the diabetic heart, causing cellular lipotoxicity and the initiation of cardiac dysfunction [30]. In the present study, despite no obvious increase in FFAs, differential expression of all eight long-chain hydroxyacylcarnitine and six long-chain acylcarnitines was detected. In addition, two TGs and three DGs were obviously upregulated in the diabetic heart. These cytotoxic lipids cause mitochondrial injury resulting in decreased ATP production and increased generation of reactive oxygen species (ROS); thereby, resulting in cardiac dysfunction in diabetic mice [11, 31]. Moreover, under these conditions lipotoxicity also causes increased expression of UCP2 and UCP3, which contributes to decline in ATP content in the diabetic heart by uncoupling oxidative phosphorylation [32, 33].

In addition to mitochondrial β-oxidation, here, peroxisomal FA β-oxidation was also found to be elevated in the diabetic heart. The majority of FAs that undergo oxidation in cardiac tissue are mono- or polyunsaturated and not saturated [34]. The decrease in PUFA observed in STZ-induced insulin deficiency is caused by a reduction in the delta 6-desaturase activity required to convert long-chain fatty acids to PUFAs [35]. Furthermore, peroxisomal β-oxidation is negatively correlated with the PUFA content in diabetic heart [9]. In accordance with our previous study, our present results show enhanced peroxisomal β-oxidation activity accompanied by deficiencies of the very long chain mono- or polyunsaturated FAs, especially EPA (C20:5n–3) and DHA (C22:6n–3), in the heart of diabetic mice. Furthermore, peroxisomal FA β-oxidation elevation promotes the production of toxic ROS. Therefore, the increased peroxisomal β-oxidation exacerbates the mitochondrial injury and impaired heart function in diabetic mice by producing ROS and reducing the important PUFAs, especially EPA and DHA [8, 36].

It has been shown previously that lipotoxicity induces the breakdown of phospholipids in rat cardiomyocytes and ultimately leads to cell death [37]. Under the conditions of lipotoxicity described here, more than half of all the differentially expressed phospholipid species detected in this study were found to be downregulated in the cardiac tissue of diabetic mice, with the exception of all the SM species and half of the PI species. Phospholipids are important biomembrane components and signaling molecules [38, 39]. A reduction in phospholipids results in mitochondrial abnormalities [40–42] and cardiac dysfunction [43, 44]; therefore, the downregulation in phospholipids in the diabetic heart could also contribute to mitochondrial cristate fusion, reduced ATP production and cardiac dysfunction. Moreover, the sphingolipid metabolic pathway was constructed in the diabetic heart.

## Conclusion

Our lipidomics study revealed distinct differences in lipid metabolism and endogenous metabolites were identified in the cardiac tissue of STZ-induced diabetic mice compared with control mice. Eight long-chain hydroxyl- and six acyl-carnitines as well as two TGs and three DGs were shown to be upregulated in the cardiac tissue of diabetic mice. In contrast, PUFAs, such as EPA, and DHA, and many phospholipids, especially MS, were downregulated with the increase in peroxisomal β-oxidation in the diabetic heart. Thus, our data suggest that the increase in toxic lipid species and decreased PUFAs, combined with the reduction in phospholipids cause mitochondrial injury and subsequent uncoupling of phosphorylation and ATP deficiency; thereby leading to diabetic heart dysfunction.

## Abbreviations

AOX1: Acyl-CoA oxidase 1
CKMB: Creatine kinase isoenzyme-MB
CPT-1α: Palmitoyl transferase 1α
DBP: D-bifunctional protein
DG: Diradylglycerol
EF: Ejection fraction
FABP3: Fatty acid binding protein 3
FAs: Fatty acids
FFA: Free fatty acid
FS: Fractional shortening
GLUT4: Glucose transporter 4
HCY: Homocysteine
HE: Hematoxylin-eosin
Hs-crp: High sensitivity C reactive protein
LDH: Lactate dehydrogenase
LV: Left ventricular
LVEDD: LV end-diastolic diameter
LVESD: LV end-systolic diameter
Lyso: Lysoglycerophospholipids
MCAD: Medium chain acyl-CoA dehydrogenase
Myo: Myoglobin
PA: Phosphatidic acid
PC: Phosphocholine
PCA: Principal component analysis
PCR: Polymerase Chain Reaction
PE: Phosphoethanolamine
PG: Phosphatidylglycerol
PI: Phosphatidylinositol
PLS-DA: Partial least squares discriminant analysis
PS: Phosphoserine
PUFA: Polyunsaturated fatty acid
ROS: Reactive oxygen species
SM: Sphinogomyelin
STZ: Streptozotocin
T-chol: Total cholesterol
T-CK: Total creatine kinase
T-FFA: Total free fatty acid
TG: Triradylglycerol
THLA: 3-Ketoacyl-CoA thiolaseA
UCP: Uncouple protein
α-HBDH: α-hydroxybutyrate dehydrogenase

## References

[1] Ji L, Liu YY, Zhang Y, Chang WG, Gong JL, Wei SN, Li XD, Qin L (2016). The antioxidant edaravone prevents cardiac dysfunction by suppressing oxidative stress in type 1 diabetic rats and in high-glucose-induced injured H9c2 cardiomyoblasts. Can J Physiol Pharmacol.

[2] Huxley RR, Peters SA, Mishra GD, Woodward M (2015). Risk of all-cause mortality and vascular events in women versus men with type 1 diabetes: a systematic review and meta-analysis. Lancet Diabetes Endocrinol.

[3] Nathan DM (2015). Diabetes advances in diagnosis and treatment. JAMA.

[4] Isfort M, Stevens SC, Schaffer S, Jong CJ, Wold LE (2014). Metabolic dysfunction in diabetic cardiomyopathy. Heart Fail Rev.

[5] Roger HU, Gregory OC, Philipp ES (2010). Lelio or. Lipid homeostasis,lipotoxicity and the metabolic syndrome. Biochimic Biophysica Acta.

[6] Fillmore N, Mori J, Lopaschuk GD (2014). Mitochondrial fatty acid oxidation alterations in heart failure, ischaemic heart disease and diabetic cardiomyopathy. Br J Pharmacol.

[7] Katsuyuki S, Hiroshi F, Tadashi Y, Jun S, Tohru I, Akira S, Teruo O (1995). Tissue-specific suppression of aortic fatty-acid-binding protein in streptozotocin-induced diabetic rats. EurJBiochem.

[8] Sharma S, Adrogue JV, Golfman L, Uray I, Lemm J, Youker K, Noon GP, Frazier OH, Taegtmeyer H (2004). Intramyocardial lipid accumulation in the failing human heart resembles the lipotoxic rat heart. FASEB J.

[9] Hou LG, Yao M, Lu X, Fang LJ, He TB, Jiang LL (2012). Reduction of n-3 PUFAs , specifically DHA and EPA , and enhancement of peroxisomal beta-oxidation in type 2 diabetic rat heart, Cardiovasc. Diabetol.

[10] Eldor R, Norton L, Fourcaudot M, Galindo C, DeFronzo RA, Abdul GM (2017). Increased lipid availability for three days reduces whole body glucose uptake, impairs muscle mitochondrial function and initiates opposing effects on PGC-1α promoter methylation in healthy subjects. PLoS One.

[11] Liepinsh E, Makrecka-Kuka M, Volska K, Kuka J, Makarova E, Antone U, Sevostjanovs E, Vilskersts R, Strods A, Tars K, Dambrova M (2016). Long-chain acylcarnitines determine ischaemia / reperfusion-induced damage in heart mitochondria. Biochem J.

[12] Kratky D, Obrowsky S, Kolb D, Radovic B (2014). Pleiotropic regulation of mitochondrial function by adipose triglyceride lipase-mediated lipolysis. Biochimie.

[13] Elezaby A, Sverdlov AL, Tu VH, Soni K, Luptak I, Qin F, Liesa M, Shirihai OS, Rimer J, Schaffer JE, Colucci WS, Miller EJ (2015). Mitochondrial remodeling in mice with cardiomyocyte-specific lipid overload. J Mol Cell Cardiol.

[14] Han XL, Yang JY, Yang K, Zhao ZD, Abendschein DR, Gross RW (2007). Alterations in myocardial cardiolipin content and composition occur at the very earliest stages of diabetes: a shotgun lipidomics study. Biochemistry.

[15] Han XL, Yang JY, Cheng H, Yang K, Abendschein DR, Gross RW (2005). Shotgun lipidomics identifies cardiolipin depletion in diabetic myocardium linking altered substrate utilization with mitochondrial dysfunction. Biochemistry.

[16] Seth A, Steel JH, Nichol D, Pocock V, Kumaran MK, Fritah A, Mobberley M, Ryder TA, Rowlerson A, Scott J, Poutanen M, White R, Parker M (2007). The transcriptional corepressor RIP140 regulates oxidative metabolism in skeletal muscle. Cell Metab.

[17] Ejsing CS, Sampaio JL, Surendranath V, Duchoslav E, Ekroos K, Klemm RW, Simons K, Shevchenko A (2009). Global analysis of the yeast lipidome by quantitative shotgun mass spectrometry. Proc Natl Acad Sci U S A.

[18] Nie H, Liu R, Yang Y, Bai Y, Guan Y, Qian D, Wang T, Liu H (2010). Lipid profiling of rat peritoneal surface layers by online normal- and reversed-phase 2D LC QToF-MS. J Lipid Res.

[19] Poornima IG, Parikh P, Shannon RP (2006). Diabetic cardiomyopathy: the search for a unifying hypothesis. Circ Res.

[20] Fang ZY, Prins JB, Marwick TH (2004). Diabetic cardiomyopathy : evidence , mechanisms , and the rapeutic implications. Endocr Rev.

[21] Tahiliani AG, Mcneill JH (1986). Diabetes-induced abnormalities in the myocardium. Life Sci.

[22] Westermann D, Walther T, Savvatis K, Escher F, Sobirey M, Riad A, Bader M, Schultheiss HP, Tschöpe C (2009). Gene deletion of the kinin receptor B1 attenuates cardiac inflammation and fibrosis during the development of experimental diabetic cardiomyopathy. Diabetes.

[23] Okamoto MM, Sumida DH, Carvalho CR, Vargas AM, Heimann JC, Schaan BD, Machado UF (2004). Changes in dietary sodium consumption modulate GLUT4 gene expression and early steps of insulin signaling. Am J Physiol Regul Integr Comp Physiol..

[24] Stenbit AE, Tsao TS, Li J, Burcelin R, Geenen DL, Factor SM, Houseknecht K, Katz EB, Charron MJ (1997). GLUT4 heterozygous knockout mice develop muscle insulin resistance and diabetes. Nat Med.

[25] Glatz JF, van Breda E, Keizer HA, de Jong YF, Lakey JR, Rajotte RV, Thompson A, van der Vusse GJ, Lopaschuk GD (1994). Rat heart fatty acid-binding protein content is increased in experimental diabetes. Biochem Biophys Res Commun.

[26] Furuhashi M, Hotamisligil GS (2008). Fatty acid-binding proteins: role in metabolic diseases and potential as drug targets. Nat Rev Drug Discov.

[27] Lopaschuk GD, Folmes CD, Stanley WC (2007). Cardiac energy metabolism in obesity. Circ Res.

[28] Amrutkar M, Cansby E, Chursa U, Nuñez-Durán E, Chanclón B, Ståhlman M, Fridén V, Mannerås-Holm L, Wickman A, Smith U, Bäckhed F, Borén J, Howell BW, Mahlapuu M (2015). Genetic disruption of protein kinase STK25 ameliorates metabolic defects in a diet-induced type 2 diabetes model. Diabetes.

[29] Carley AN, Severson DL (2005). Fatty acid metabolism is enhanced in type 2 diabetic hearts. Biochim Biophys Acta.

[30] Van de Weijer T, Schrauwen-Hinderling VB, Schrauwen P (2011). Lipotoxicity in type 2 diabetic cardiomyopathy. Cardiovasc Res.

[31] Tonin AM, Grings M, Busanello EN, Moura AP, Ferreira GC, Viegas CM, Fernandes CG, Schuck PF, Wajner M (2010). Long-chain 3-hydroxy fatty acids accumulating in LCHAD and MTP deficiencies induce oxidative stress in rat brain. Neurochem Int.

[32] Brand MD, Affourtit C, Esteves TC, Green K, Lambert AJ, Miwa S, Pakay JL, Parker N (2004). Mitochondrial superoxide: production, biological effects, and activation of uncoup. Free Radic Biol Med.

[33] Brand MD, Esteves TC (2005). Physiological functions of the mitochondrial uncoupling proteins UCP2 and UCP3. Cell Metab.

[34] Ovide-Bordeaux S, Grynberg A (2004). Docosahexaenoic acid affects insulin deficiency-and insulin resistance-induced alterations in cardiac mitochondria. Am J Physiol Regul Integr Comp Physiol.

[35] Lopaschuk GD, Ussher JR, Folmes CD, Jaswal JS, Stanley WC (2010). Myocardial fatty acid metabolism in health and disease. Physiol Rev.

[36] Shi Y, Sun X, Sun Y, Hou L, Yao M, Lian K, Li J, Lu X, Jiang L (2016). Elevation of cortical C26:0 due to the decline of peroxisomal β-oxidation potentiates amyloid β generation and spatial memory deficits via oxidative stress in diabetic rats. Neuroscience.

[37] Janero DR, Burghardt B, Lopez R (1988). Protection of cardiac membrane phospholipid against oxidative injury by calcium antagonists. Biochem Pharmacol.

[38] Merrill A.H., Schmelz E-M., Dillehay D.L., Spiegel S., Shayman J.A., Schroeder J.J., Riley R.T., Voss K.A., Wang E. (1997). Sphingolipids—The Enigmatic Lipid Class: Biochemistry, Physiology, and Pathophysiology. Toxicology and Applied Pharmacology.

[39] Okazaki T (1989). Sphingomyelin turnover induced by vitamin D3 in HL-60 cells. Role in cell differentiation. J Biol Chem.

[40] Pollard AK, Ortori CA, Stöger R, Barrett DA, Chakrabarti L (2017). Mouse mitochondrial lipid composition is defined by age in brain and muscle. Aging.

[41] Chan EY, McQuibban GA (2012). Phosphatidylserine decarboxylase 1 ( Psd1 ) promotes mitochondrial fusion by regulating the biophysical properties of the mitochondrial membrane and alternative topogenesis of mitochondrial genome maintenance protein. J Biol Chem.

[42] Puurunen J, Sulkama S, Tiira K, Araujo C, Lehtonen M, Hanhineva K, Lohi H (2016). A non-targeted metabolite profiling pilot study suggests that tryptophan and lipid metabolisms are linked with ADHD-like behaviours in dogs. Behav Brain Funct.

[43] Birse RT, Bodmer R (2015). Lipotoxicity and cardiac dysfunction in mammals and drosophila. Crit Rev Biochem Mol Biol.

[44] Lim HY, Wang W, Wessells RJ, Ocorr K, Bodmer R (2011). Phospholipid homeostasis regulates lipid metabolism and cardiac function through SREBP signaling in drosophila. Genes Dev.

